# Frontal plane sloped support surfaces: their effect on body balance and posture organization

**DOI:** 10.3389/fnhum.2026.1705734

**Published:** 2026-03-04

**Authors:** Alain Hamaoui, Siripatra Atsawakaewmongkhon

**Affiliations:** 1 CIAMS, Université Paris Saclay, Orsay, France; 2 Department of Physical Therapy, School of Integrative Medicine, Mae Fah Luang University, Chiang Rai, Thailand

**Keywords:** articular angles, body balance, EMG, lower limbs, sloped surface

## Abstract

**Introduction:**

Sloped surfaces are common in daily life, as ground surfaces are frequently inclined for purposes such as accessibility, transportation, and drainage. This study assessed how support surfaces inclined in the frontal plane might affect body balance and posture.

**Methods:**

Fifteen subjects underwent a posturographic examination with the support surface sloped at 0°, 7°, and 15°. The tests were associated with goniometric measurements of knees and hips, and surface EMG of the Tibialis Anterior, Soleus, Rectus Femoris, and Biceps Femoris.

**Results:**

It has been shown that standing on a lateral sloped surface shifts the CP toward the direction of the slope and increases the CP velocity and mean displacement. Goniometric data revealed that the knees and ankles were flexed on the contralateral side of the slope, with a higher activity of the Rectus Femoris and Tibialis Anterior.

**Discussion:**

From the main findings, it was assumed that surfaces inclined in the frontal plane result in poorer body balance and increased fall risk. This might have significant implications in rehabilitation and accessibility.

## 1 Introduction

Understanding the mechanisms that provide humans with the ability to maintain a standing posture has been a topic of scientific research for centuries. A significant step was taken in the middle of the twentieth century, using force plates to assess body balance through the position and displacements of the center of pressure (CP) (Hellebrandt, 1938; Thomas and Whitney, 1959). These seminal studies, which were performed in a natural standing posture, were quickly followed by experiments exploring body balance in more challenging postures such as sustained leaning (Murray et al., 1975; Whitney, 1962), eyes closed (Stribley et al., 1974), or narrowed base of support (Day et al., 1993; Goldie et al., 1989; Winter et al., 1996). It was only in the 2000s that assessing body balance on sloped surfaces began, initially to prevent the risk of falls in roofers (Atsawakaewmongkhon et al., 2024; Baldridge and King, 2022; Choi, 2008; Dutt-Mazumder et al., 2016; King et al., 2020; Lin et al., 2012; Mezzarane and Kohn, 2007; Simeonov et al., 2003). Most of these works explored the effect of sloped surfaces in the sagittal plane, the most frequently encountered in daily life, although a simple 90° turn of the body moves the slope from sagittal to frontal.

A frontal sloped surface is expected to induce a triple flexion of the ankle, knee, and hip at the contralateral side (i.e., opposite to the side of the slope) to keep the pelvis horizontal. This new angular position would then require an increased activity of the lower limb extensors. In terms of postural control, we might predict a shift of the CP toward the side of the slope, and an increased postural sway along the medial-lateral (ML) axis. Taken together, these effects might be more challenging for body balance than the variations observed for surfaces sloped in the sagittal plane (Atsawakaewmongkhon et al., 2024).

To our knowledge, only two studies have focused on the effect of lateral slopes on postural balance (Lin and Nussbaum, 2012; Simeonov et al., 2009), and both showed an increase in CP displacements. However, neither included the goniometric or EMG measurements needed to explore the adaptation mechanisms. In addition, no data on the CP mean position were presented, leaving the question of weight transfer unanswered.

The main goal of this study was to assess the effect of sloped surfaces in the frontal plane on body balance and posture. The experimental paradigm consisted of a posturographic examination carried out with different directions of surface slope and angles, as well as goniometric and EMG examinations of the lower limbs.

## 2 Method

### 2.1 Participants

Fifteen healthy adults (7 males and 8 females; age: 26.64 ± 2.45 years old; weight: 74.91 ± 20.58 kg; height: 168.09 ± 6.48 cm; body mass index: 22.60 ± 2.00 kg/m^2^), participated in the experiment. All subjects were free of any pathology or medical treatment that might affect balance control or ankle joint mobility (inclusion checklist). The experimental protocol was approved by the local ethics committee, CER Paris-Saclay, with the reference CER-Paris-Saclay-2023-497. Written informed consent was obtained from each participant, in accordance with the declaration of Helsinki.

### 2.2 Material

#### 2.2.1 Force plate and slant board

A six-channel force plate (AMTI-OR6, Watertown, United States), recording the ground reaction forces and moments acting on the top surface was used to calculate the coordinates of the CP along the anterior–posterior (X) and the medial-lateral (Y) axes. The positive direction of X, Y and Z axes was oriented forward, leftward, and downward, respectively.

**Figure 1:**
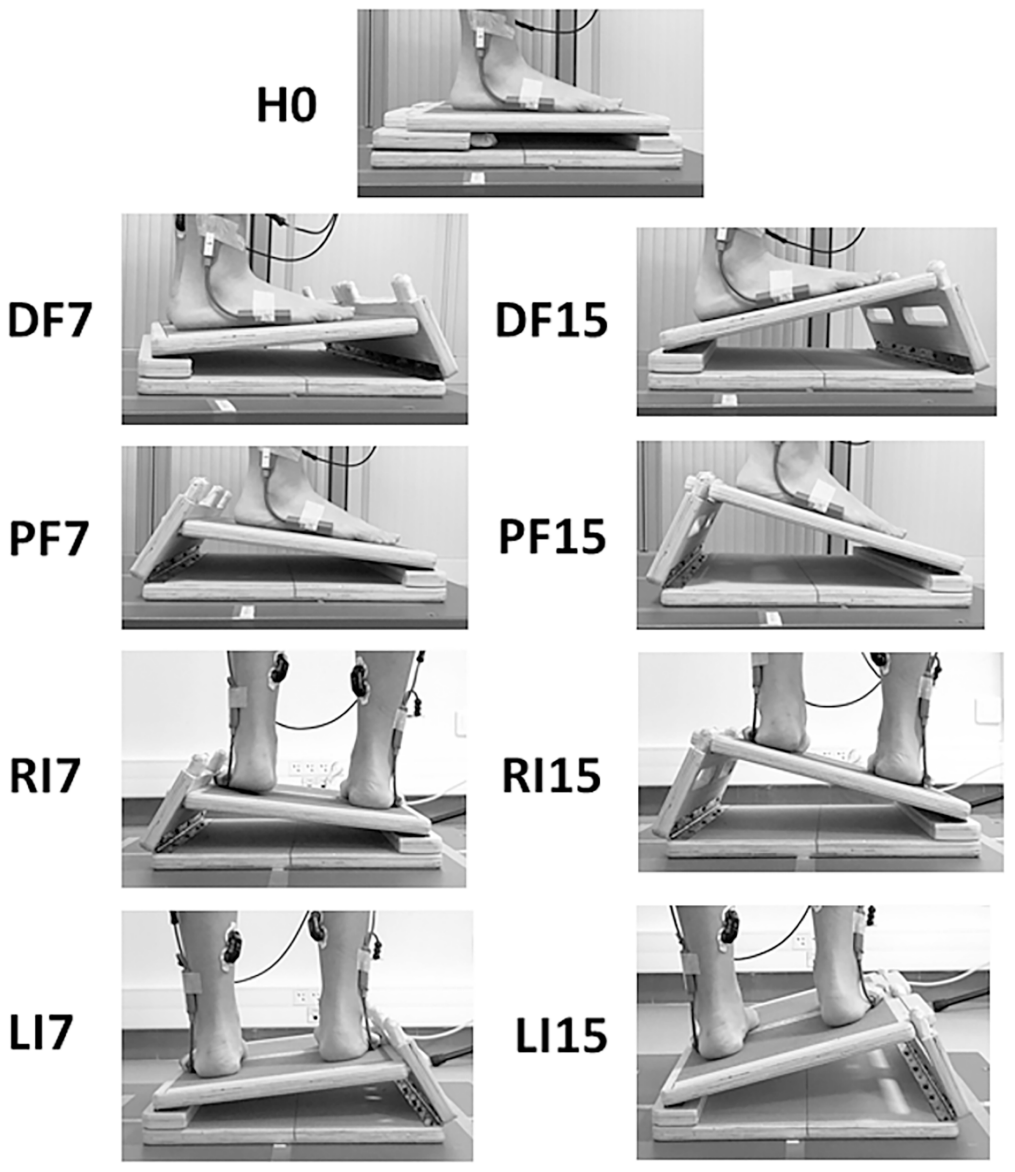
Slant board mounted on the force plate in different experimental conditions. Horizontal (H0, reference condition), inclination towards the right side at 7° (RI7) and 15° (RI15), inclination towards the left side at 7° (LI7) and 15° (LI15), backward inclination at 7° (DF7) and 15° (DF15), forward inclination at 7° (PF7) and 15° (PF15).

An adjustable and customized slant board (43 × 33 × 5.5 cm) was mounted on the force plate and used to set the support surface in the sagittal and frontal planes at three different slope angles (0°, 7°, and 15°). It was made of wood and covered with sandpaper to prevent slipping (Figure 1). Slopes of 7° and 15° were selected to enable comparisons with existing studies that used angles greater than or equal to 10° (Lin et al., 2012; Simeonov et al., 2009) and to test a condition closer to the lower angles typically encountered in daily activities.

Once the slant board has been attached using double-sided tape, an offset was performed in the reference H0 condition (surface slope at 0°).

The coordinates of the CP are calculated from the following formulas:


$$x_{p}=\frac{-hF_{x}+M_{y}}{F_{z}}$$

$$y_{p}=\frac{-hF_{y}-M_{x}}{F_{z}}$$

with:

Xp: CP along the anterior–posterior axis;
Yp: CP along the medial-lateral axis;
h: distance between the center of the slant board and the origin of the force plate;
Fx: reaction force of the ground along the anterior–posterior axis;
Fy: reaction force of the ground along the medial-lateral axis;
Fz: ground reaction force along the vertical axis;
Mx: moment along the anterior–posterior axis;
My: moment along the medial-lateral axis.

#### 2.2.2 Surface electromyogram (sEMG)

A 16-channel wireless EMG device (Pico EMG model, Cometa, Milan, Italy) was used to assess the surface electrical activities of four main flexors/extensors of the ankles and knees at the right and left sides: tibialis anterior (TA), Soleus (Sol), Rectus femoris (RecF) and Biceps femoris (BiF). The focus was placed on these muscles because we expected adaptations to lateral sloped surfaces to consist primarily of lower-limb flexion and extension, compensating for the height difference between the two lateral sides. The participants’ skin was shaved when needed, abraded, and cleaned with alcohol to reduce skin impedance to below 5 kΩ. 10-mm diameter (conductive area) Ag/AgCl pre-gelled disposable surface electrodes (H124SG KendallTM) were applied in a bipolar configuration over the muscle belly parallel to the direction of the muscle fibers. The inter-electrode distance was 20 mm. The electrode placement followed the recommendations of Barbero et al. (2012), with the additional use of palpation and manual resistance tests proposed by the SENIAM (Hermens et al., 2000).

To allow for normalization of the EMG signals by their maximum values, two 3-s isometric MVCs were performed for each muscle, with an inter-trial 30-s resting time, prior to the posturographic examination.

#### 2.2.3 Electrogoniometers

Four twin axis goniometers, which permit the simultaneous measurement of angles in two planes (SG150 and SG110, Biometrics Ltd.) were used to measure the angular position of the ankles and knees in flexion/extension during the posturographic examination. The goniometers were positioned using imaginary lines passing through the following anatomical landmarks: the lateral surface of the greater trochanter, the epicondyle of the femur, the lateral surface of the lateral malleolus, and the lateral surface of the base of the fifth metatarsal.

#### 2.2.4 Data acquisition system

The data collected by the force plate, the EMG system, and the electrogoniometers were digitized at 1000 Hz using an A/D converter (CompactDAQ, National Instruments, Austin, United States) controlled by a custom program using graphical programming (LabVIEW software, National Instruments, Austin, United States).

### 2.3 Procedure

Subjects stood barefoot on the force plate equipped with the slant board, with their arms hanging at the sides and their feet apart at hip width (visually assessed by the experimenter). The transverse line connecting the two ankles was positioned in alignment with the transverse axis of the force plate. They were asked to stand as still as possible and to keep their gaze fixed on a 5-cm black disc placed at their eye level on a wall located 1.5 m in front of them. Four 30 s trials were performed under nine experimental conditions in the sagittal and frontal planes: horizontal 0° (H0), inclined towards the right side at 7° (RI7) and 15° (RI15), inclined toward the left side at 7° (LI7) and 15° (LI15), inclined backward (feet in dorsal flexion) at 7° (DF7) and 15° (DF15), inclined forward (feet in plantar flexion) at 7° (PF7) and 15° (PF15). Forward and backward inclination conditions were implemented to enable comparison of the effects of support surface inclination between the frontal and sagittal planes.

After a training period, the recording started with condition H0 which was considered as the reference posture. The other conditions were randomly assigned to avoid any order effect. The rest time was 20s between trials and 2 min between conditions.

### 2.4 Data analysis

#### 2.4.1 Posturographic data

Six classical posturographic calculated in the time domain were used as indicators of body balance:

- X0, Y0: mean position of the CP along the anterior–posterior (X0) and medial-lateral (Y0) axes;
- Xm, Ym: mean deviation of the CP along the anterior–posterior (Xm) and medial-lateral (Ym) axes;
- Vm-ap, Vm-ml: mean velocity along the anterior–posterior (Vm-ap) and medial-lateral axes.

#### 2.4.2 Normalized EMG

The mean rectified EMGs were calculated from each recording (sum of the absolute values of all signal points divided by the number of points), before being normalized by the data calculated in the MVC condition. No filtering was used.

#### 2.4.3 Electrogoniometric data

For each goniometer, the mean value was calculated from each recording, providing data on the mean articular position in flexion/extension of the two ankles and two knees.

#### 2.4.4 Statistical analysis

Statistical analysis was performed using JASP Statistics software (version 0.18.3.0, The Netherlands). A one-way repeated-measures analysis of variance (ANOVA) was conducted with the support surface slope as a dependent variable with nine modalities (H0, RI7, RI15, LI7, LI15, DF7, DF15, PF7, and PF15). When a statistical difference was reached, a simple contrast test was used with H0 as the reference condition. Sphericity was assessed using Mauchly’s test. When the assumption of sphericity was violated (*p* < 0.05), degrees of freedom were corrected using the Greenhouse–Geisser method. The significance level was set at *p*-values <0.05.

## 3 Results

As the main objective of this study was to assess the effect of support surfaces sloped in the frontal plane, results will focus on the corresponding conditions.

### 3.1 Goniometric data

#### 3.1.1 Inclinations in the frontal plane

**Figure 2:**
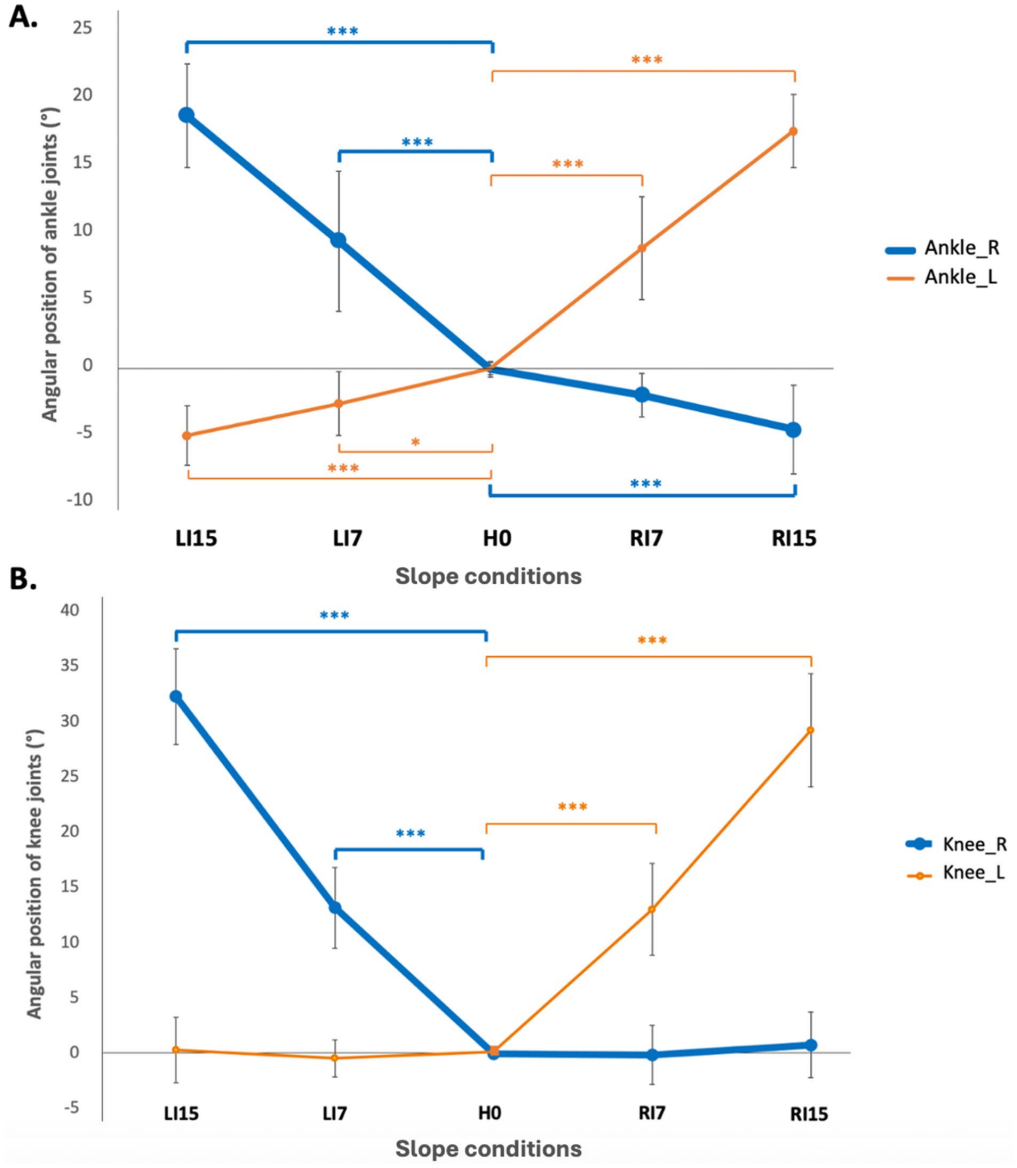
Angular position (°) of right (Ankle_R) and left (Ankle_L) ankles **(A)** and right (Knee_R) and left (Knee_L) knees **(B)** under lateral slope conditions. Mean values and standard deviations (grey rows) are presented for lateral slope conditions: horizontal (H0), inclination towards the right side at 7° (RI7) and 15° (RI15), inclination towards the left side at 7° (LI7) and 15° (LI15). Positive data represent flexion and negative data, extension (***p < 0.001).

Data analysis revealed that the knees and ankles were systematically flexed on the contralateral side, i.e., opposite to the side of the inclination (Table 1; Figure 2). This variation was highly significant for all sloped conditions compared to H0, with *p* < 0.001.

**Table 1:** Angular position (°) of ankle and knee joints under all slope conditions.

	Ankle_L (°)	*p*	Knee_L (°)	*p*	Ankle_R (°)	*p*	Knee_R (°)	*p*
H0	0.21 ± 0.6		0.1 ± 0.3		−0.2 ± 0.7		0.0 ± 0.2	
RI7	8.74 ± 5.3	***	13.0 ± 4.1	***	−2.2 ± 2.0	ns	0.8 ± 3.0	ns
RI15	17.4 ± 3.6	***	29.2 ± 5.1	***	−4.7 ± 4.1	***	−0.1 ± 2.7	ns
LI7	−2.8 ± 2.7	*	−0.5 ± 1.7	ns	9.2 ± 6.0	***	13.2 ± 3.6	***
LI15	−5.2 ± 3.1	***	0.3 ± 3.0	ns	18. 5 ± 4.5	***	32.3 ± 4.3	***
DF7	5.8 ± 1.5	***	1.6 ± 1.7	ns	6.2 ± 1.2	***	1.0 ± 1.9	ns
DF15	14.0 ± 2.2	***	5.7 ± 3.7	*	16.1 ± 2.1	***	4.5 ± 3.4	**
PF7	−6.5 ± 2.0	***	−0.2 ± 0.8	ns	−5.5 ± 1.1	***	0.8 ± 1.1	ns
PF15	−12.5 ± 2.2	***	0.8 ± 1.6	ns	−11.2 ± 1.7	***	1.8 ± 1.9	ns

Mean values ± standard deviations are presented for all slope conditions: horizontal (H0), inclination toward the right (RI7, RI15) and left sides (LI7, LI15), inclination backward (DF7, DF15) and forward (PF7, PF15). Positive values represent flexion and negative data extension. *p < 0.05, **p < 0.01, ***p < 0.001 and ns = non-significant.

#### 3.1.2 Inclinations in the sagittal plane

A dorsal flexion was systematically observed in backward slopes and a plantar flexion in forward slopes (*p* < 0.001 for all conditions compared to H0) (Table 1). Unexpectedly, a significant increase in knee flexion of about 5° was also found in DF15 (Table 1).

### 3.2 EMG data

#### 3.2.1 Inclinations in the frontal plane

**Figure 3:**
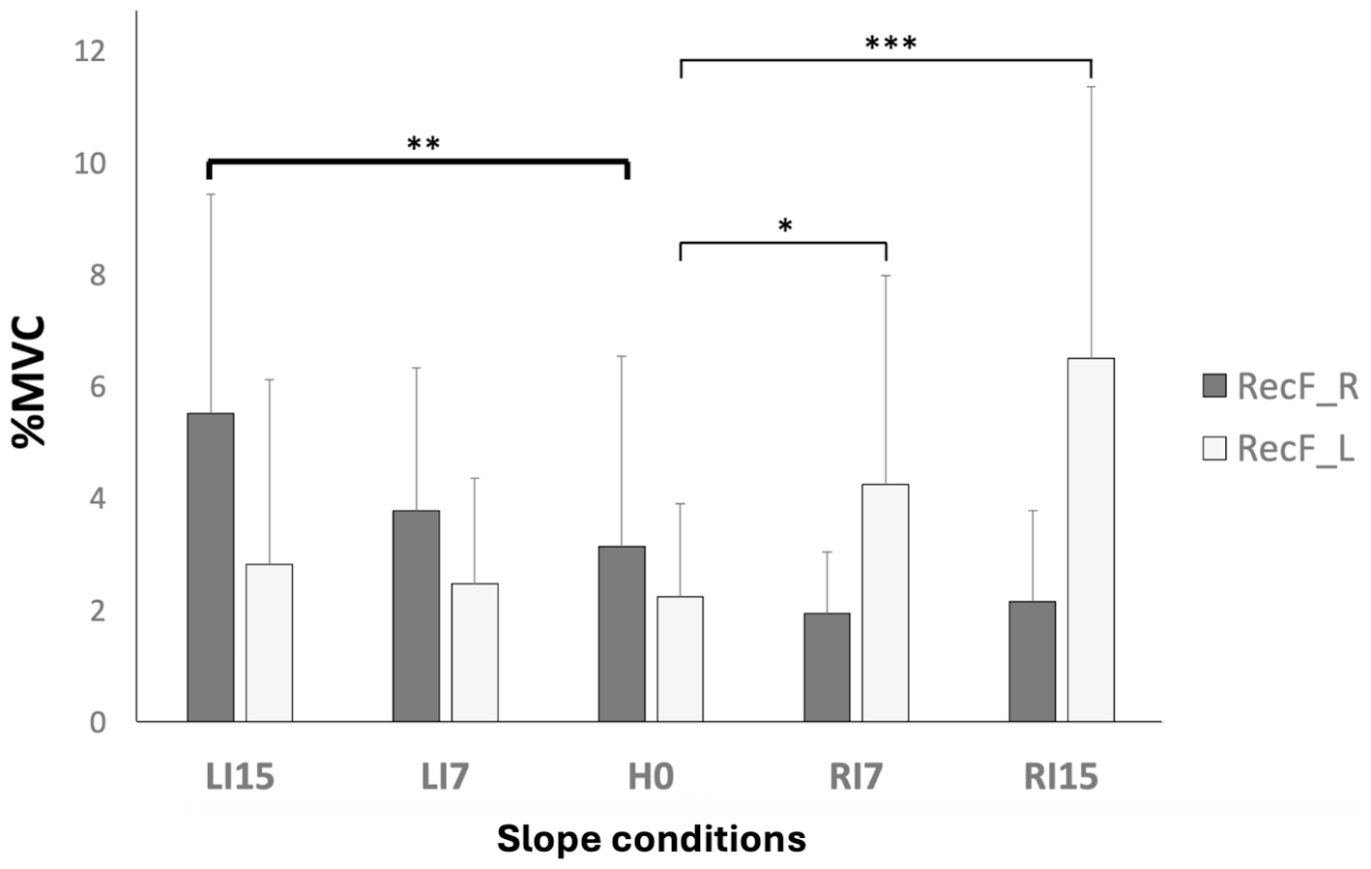
Normalized EMG (%) of rectus femoris. EMGs of the right (RecF-R) and left (RecF-L) rectus femoris muscle are presented in each condition of lateral slope: horizontal (H0), inclination towards the right side at 7° (RI7), and 15° (RI15), inclination towards the left side at 7° (LI7), and 15° (LI15). Mean values ± standard deviations are expressed as a percentage of the mean value obtained in the MVC condition (*p < 0.05, **p < 0.01, ***p < 0.001).

For ankle muscles, a significant increase was observed for the contralateral TA in LI15 (*p* < 0.01) and RI15 conditions (*p* < 0.001), while Sol only displayed a significant decrease in RI15 at the left side (*p* < 0.001) (Table 2). For knee muscles, an increase of the RecF was observed on the contralateral side of the inclination, with significant variations on the right side in LI15 (*p* < 0.01) and on the left side in RI7 (*p* < 0.05) and RI15 (*p* < 0.001) (Figure 3).

**Table 2:** Normalized EMG (% of the MVC) under all slope conditions.

	TA_R	*p*	TA_L	*p*	Sol_R	*p*	Sol_L	*p*	RecF_R	*p*	RecF_L	*p*	BiF_R	*p*	BiF_L	*p*
	(% MVC)		(%MVC)		(% MVC)		(% MVC)		(% MVC)		(% MVC)		(% MVC)		(% MVC)	
H0	2.46 ± 2.13		2.59 ± 2.69		13.40 ± 4.76		14.83 ± 4.91		3.14 ± 3.41		2.25 ± 1.67		3.64 ± 2.32		3.64 ± 2.89	
RI7	1.77 ± 1.25	ns	1.94 ± 1.24	ns	14.05 ± 4.96	ns	12.08 ± 4.47	ns	1.95 ± 1.10	ns	4.26 ± 3.74	*	2.99 ± 2.05	ns	4.07 ± 3.20	ns
RI15	2.20 ± 1.62	ns	6.15 ± 6.96	**	14.05 ± 7.74	ns	9.48 ± 4.65	***	1.17 ± 1.63	ns	6.52 ± 4.85	***	2.80 ± 1.32	ns	3.09 ± 2.25	ns
LI7	3.14 ± 5.01	ns	1.69 ± 0.94	ns	12.26 ± 6.20	ns	12.26 ± 4.46	ns	3.79 ± 2.55	ns	2.48 ± 1.89	ns	3.08 ± 1.52	ns	3.24 ± 1.98	ns
LI15	6.00 ± 9.00	**	2.00 ± 0.97	ns	10.80 ± 5.55	ns	13.27 ± 6.29	ns	5.52 ± 3.93	**	2.83 ± 3.30	ns	4.20 ± 2.91	ns	3.17 ± 1.89	ns
DF7	3.32 ± 4.86	ns	2.69 ± 3.67	ns	12.81 ± 5.11	ns	13.31 ± 4.87	ns	2.55 ± 1.56	ns	2.85 ± 1.92	ns	3.34 ± 2.03	ns	3.62 ± 2.54	ns
DF15	15.10 ± 5.55	***	15.64 ± 6.00	***	10.25 ± 4.83	*	8.78 ± 4.96	***	4.89 ± 2.98	**	6.54 ± 6.01	***	4.54 ± 4.24	ns	4.50 ± 4.72	ns
PF7	2.22 ± 2.39	ns	1.57 ± 0.83	ns	16.23 ± 5.61	*	18.32 ± 6.75	*	2.06 ± 1.13	ns	2.16 ± 1.37	ns	3.78 ± 2.51	ns	4.01 ± 3.31	ns
PF15	1.78 ± 0.84	ns	1.92 ± 1.06	ns	22.30 ± 8.31	***	25.63 ± 9.00	***	2.11 ± 1.09	ns	2.74 ± 2.07	ns	3.48 ± 2.00	ns	4.08 ± 2.55	ns

EMGs of right (R) and left (L) tibialis anterior (TA), Soleus (Sol), Rectus femoris (RecF), and Biceps femoris (BiF) are presented for all slope conditions: horizontal (H0), inclination toward the right (RI7, RI15) and left sides (LI7, LI15), inclination backward (DF7, DF15) and forward (PF7, PF15). Mean values ± standard deviations are expressed as a percentage of the mean value obtained in the MVC condition. *p < 0.05, **p < 0.01, ***p < 0.001 and ns = non-significant.

#### 3.2.2 Inclinations in the sagittal plane

Data from ankle muscles showed an increased activity of TA in DF15 and of Sol in PF7 and PF15, but a lower activity of Sol was observed in DF15 on the left and right sides (Table 2). For knee muscles, significant variations were only observed for RecF with an increase in DF15 (*p* < 0.001 on the left side and *p* < 0.01 on the right side) (Table 2).

### 3.3 Posturographic data

#### 3.3.1 Inclinations in the frontal plane

**Figure 4:**
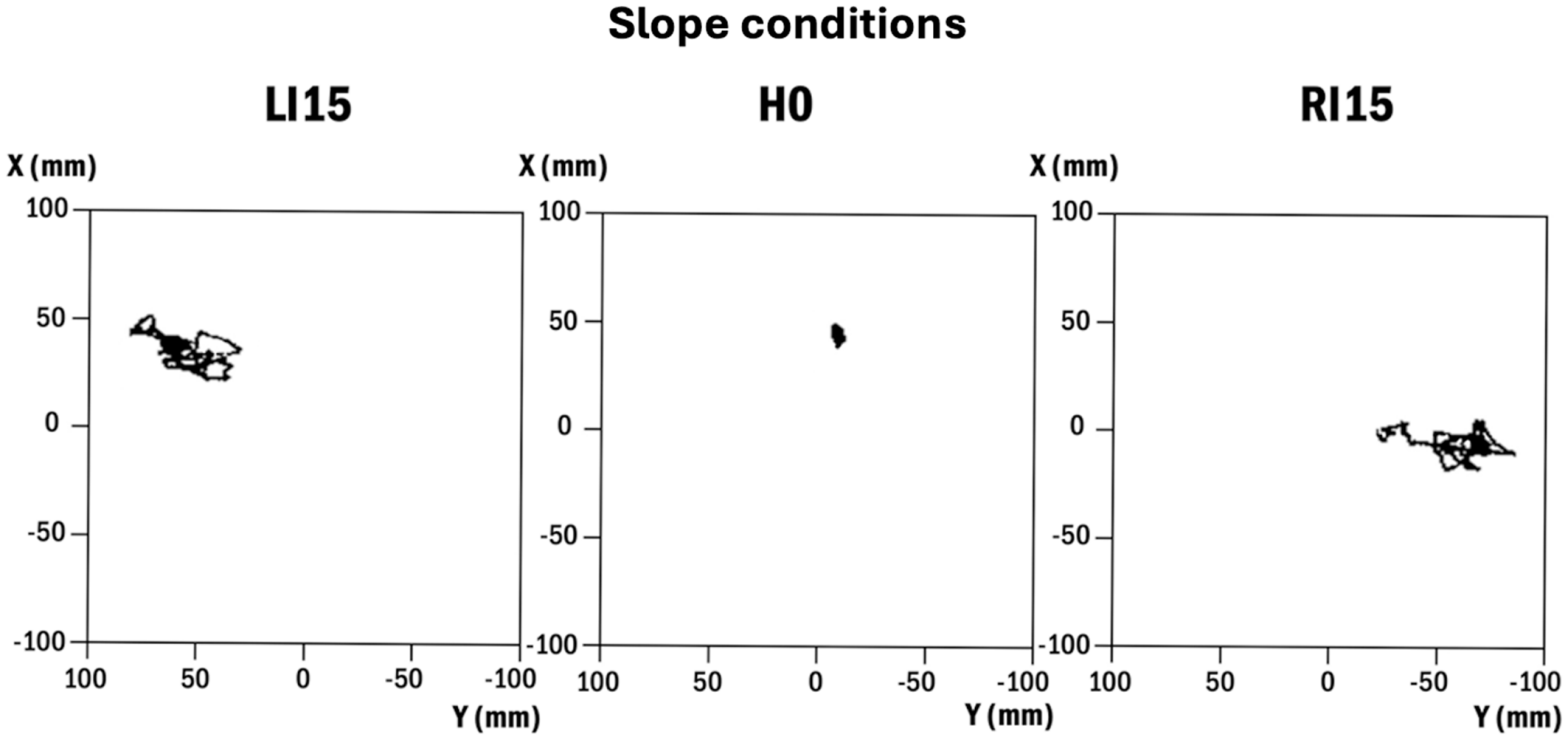
Representative traces of 30-s CP trajectory in three lateral slope conditions. From left to right: left inclination at 15° (LI15), horizontal (H0), right inclination at 15° (RI15). Y represents the medial-lateral axis and X the anterior–posterior axis.

Visual inspection of the posturograms suggested that the CP followed the direction of the inclination, with the mean position shifted to the left side in LI conditions and to the right side in RI conditions. In addition, the traces appeared larger, especially along the ML axis, when the support surface was sloped (Figure 4).

Posturographic data confirmed this impression and showed that the mean position of the CP along the ML axis (Y0) followed the direction and amplitude of the slope, with significant variations for all conditions relative to H0 (Table 3). No variation was noted along the anterior–posterior axis, with X0 remaining stable between all conditions.

**Table 3:** Posturographic parameters under all slope conditions.

	X0 (mm)	*p*	Y0 (mm)	*p*	Xm (mm)	*p*	Ym (mm)	*p*	Vm-ap (mm/s)	*p*	Vm-ml (mm/s)	*p*
H0	50.70 ± 18		−4.52 ± 6		2.98 ± 0.95		1.35 ± 0.45		244 ± 39		224 ± 31.76	
RI7	43.56 ± 20	ns	−31.97 ± 18	***	3.77 ± 1.52	**	3.14 ± 1.18	***	245 ± 36	ns	232 ± 31.68	*
RI15	44.17 ± 14	ns	−60.93 ± 18	***	3.43 ± 0.96	ns	4.69 ± 1.91	***	245 ± 38	ns	248 ± 33.76	***
LI7	43.95 ± 18	ns	14.29 ± 22	**	3.61 ± 1.17	*	3.61 ± 1.72	***	243 ± 38	ns	235 ± 34.71	**
LI15	45.84 ± 15	ns	32.77 ± 14	***	3.81 ± 1.38	**	4.37 ± 1.33	***	244 ± 38	ns	247 ± 33.46	***
DF7	50.10 ± 16.01	ns	−2.87 ± 10.1	ns	3.40 ± 1.03	ns	1.47 ± 0.44	ns	248.03 ± 36.20	ns	226.66 ± 32.32	*
DF15	47.15 ± 10.95	*	−4.63 ± 6.9	ns	4.46 ± 1.10	***	2.02 ± 0.81	***	250.83 ± 36.42	*	231.65 ± 33.03	***
PF7	59.23 ± 18.59	ns	−1.46 ± 7.3	ns	3.00 ± 0.98	ns	1.40 ± 0.48	ns	250.38 ± 39.45	*	228.05 ± 31.86	***
PF15	68.69 ± 16.36	**	−2.45 ± 7.07	ns	3.09 ± 1.09	ns	1.62 ± 0.42	ns	261.69 ± 40.84	***	231.33 ± 33.00	***

Mean values ± standard deviations are presented in all slope conditions: horizontal (H0), inclination toward the right (RI7, RI15) and left sides (LI7, LI15), inclination backward (DF7, DF15) and forward (PF7, PF15). Posturographic parameters are the mean position of the CP along the anterior–posterior (X0) and the medial-lateral (Y0) axes, the mean deviation of the CP along the anterior–posterior (Xm) and the medial-lateral (Ym) axes, and the average velocity of CP along the anterior–posterior (Vm-ap) and the medial-lateral (Vm-ml) axes. *p < 0.05, **p < 0.01, ***p < 0.001 and ns = non-significant.

**Figure 5:**
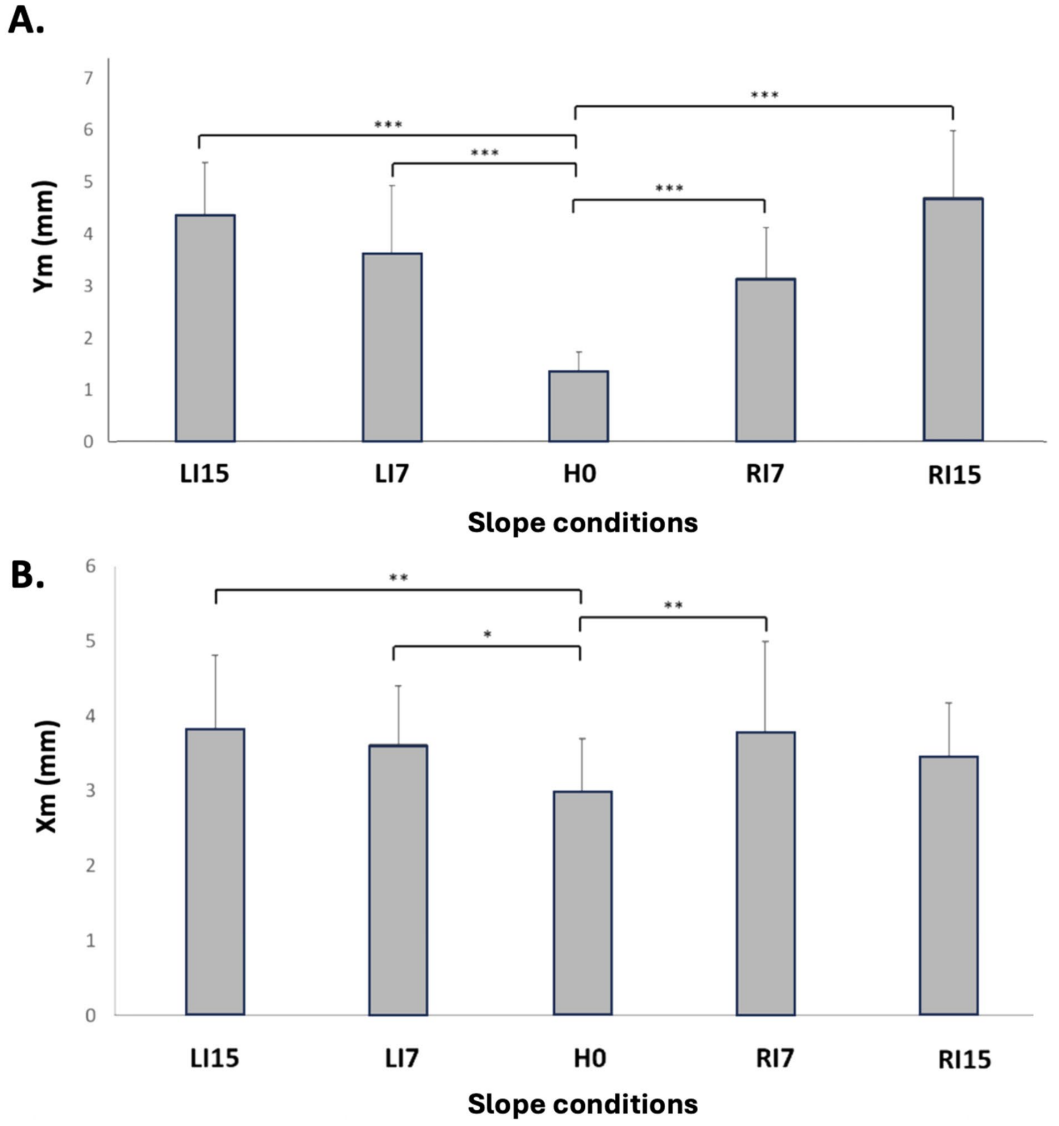
Mean deviation of the CP along the anterior-posterior axis (Xm) **(A)** and medial-lateral axis (Ym) **(B)** axes as a function of lateral surface slope. Means and standard deviations are presented in each experimental condition: horizontal (H0), inclination towards the right side at 7° (RI7) and 15° (RI15), inclination towards the left side at 7° (LI7) and 15° (LI15) (*p < 0.05, **p < 0.01, ***p < 0.001).

**Figure 6:**
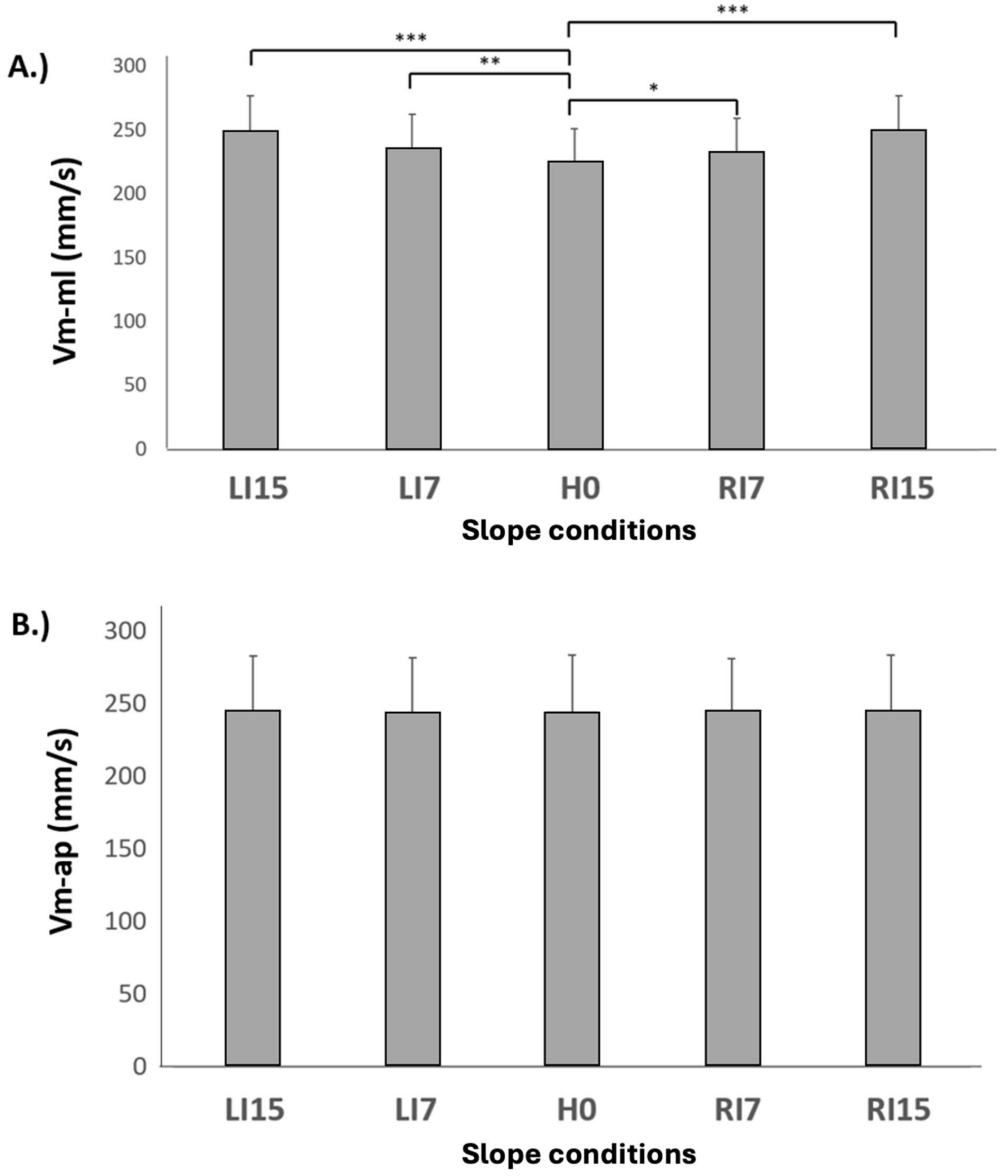
Mean velocity of the CP along the medial-lateral axis (V_m-tr_) **(A)** and anterior-posterior (V_m-ap_) axis **(B)** as a function of lateral surface slope. Means and standard deviations are presented in each experimental condition: horizontal (H0), inclination towards the right side at 7° (RI7) and 15° (RI15), inclination towards the left side at 7° (LI7) and 15° (LI15) (*p < 0.05, **p < 0.01, ***p < 0.001).

When focusing on CP displacements, the mean displacement along the medial-lateral axis (Ym) presented a significant increase in all conditions relative to H0 (Figure 5B) with *p* < 0.001. A main effect was observed along the ap-axis (Xm) (Figure 5A), but the increase was only significant in LI7 (*p* < 0.05), LI15 (*p* < 0.01), and RI7 (*p* < 0.01) (Table 3). The mean velocity along the ml axis (Vm-ml) followed the same variation as Xm, with a significant increase in all conditions relative to H0 (Figure 6A; Table 3) (*p* < 0.01 in RI7, *p* < 0.001 in RI15, *p* < 0.01 in LI7, *p* < 0.001 in LI15). However, no main effect was observed for Vm-ap, whose values were stable across the different conditions (Figure 6B).

#### 3.3.2 Inclinations in the sagittal plane

As for the lateral inclinations, the CP followed the direction of the slope, with X0 being significantly lower in DF15 (*p* < 0.05) and higher in PF15 (*p* < 0.01) (Table 3). No variation was observed for Y0. CP. Mean displacements varied the same way along the anterior–posterior (Xm) and medial-lateral (Ym) axes (Table 3), with no effect for PF7, PF15, and DF7, and an increase in DF15 (*p* < 0.001).

The mean velocity was more sensitive than the mean displacement, with Vm-ap being increased in DF15 (*p* < 0.05), PF7 (*p* < 0.05) and PF15 (*p* < 0.001) as well as Vm-ml (*p* < 0.05 in DF7, *p* < 0.001 in DF15, PF7 and PF15) (Table 3).

## 4 Discussion

### 4.1 Adaptation of lower limbs’ angular position

Under lateral slope conditions (RI and LI), flexion of the knee and ankle was observed on the contralateral side, with ankle angles approximating the surface slope inclination, likely to maintain a horizontal pelvic alignment. For slope conditions in the sagittal plane (DF and PF), a significant flexion of the knee (approximately 5°) was observed in DF15, probably to relieve the passive tension of the gastrocnemius. As bi-articular muscles crossing the knee and ankle, they are expected to be stretched by the ankle dorsal flexion, and shortened by knee flexion. These results and their interpretation seem particularly novel, since, to our knowledge, the existing literature has not provided goniometric or kinematic data of the lower limbs during stance on sloped surfaces.

### 4.2 New muscular pattern of the lower limbs

In line with our hypothesis, the variation of the lower limbs’ angular position in relation to surface slope was associated with a new pattern of muscular activity. For lateral slope conditions, the most remarkable result was an increase in the RecF activity on the contralateral side of the slope. Given that the line of gravity moves from behind the articular center in normal to forward standing (Hasegawa et al., 2017), the activity observed could be explained by the need to compensate for the increased effect of gravity on a flexed lower limb. For ankle muscles, the TA presented a significantly increased activity associated with the ankle flexion on the contralateral side (left TA in RI15 and right TA in LI15 with *p* < 0.01). This might be explained by the higher level of activity required to maintain a joint in a closed-pack position, facing higher tension of the joint’s passive tissues.

In conditions of sagittal slope (DF, PF), the analysis of thigh muscles showed a higher activity of RecF in DF15 on both sides, which can be related to the necessity of stabilizing the flexed knee. The variations in Sol and TA EMG activity were consistent with previous findings (Atsawakaewmongkhon et al., 2024; Baldridge and King, 2022), with higher normalized EMG values observed when the muscles were in a shortened position (PF7 for GasM, *p* < 0.05; PF15 for GasM, *p* < 0.001; DF15 for TA, *p* < 0.001).”

### 4.3 Postural stability under the influence of surface slope

When analyzing the posturographic data in conditions of lateral slope, it first appeared that the mean position of the CP was significantly shifted according to the direction and amplitude of the slope. This variation could be considered a mechanical effect of the slope, which moves the body’s center of gravity in its direction.

According to the study of Murray et al. (1975), the area of stability represented by CP displacements in maximal voluntary body tilt equals 54% of the base of support in the anterior–posterior axis and 59% in the medial-lateral axis. For a male foot breadth of 9.9 cm (Hawes and Sovak, 1994), this represents a length of 5.8 cm in the ML axis. A CP shift that reaches more than 5 cm in the current study (RI15 condition), would then place the body very close to the limits of falling. When considering the sloped surface in the sagittal plane (DF and PF conditions), the CP shift was lower (about 1.2 cm) and the stability area was larger. As a consequence, the CP remains more distant from the borders of the stability area, with a lower risk of falls.

When considering CP displacements in lateral slope conditions, mean displacements and velocity were increased, especially along the ML axis. These results are in line with the study of Lin et al. (2012) who found higher CP Vm-ml and sway area when the participants were standing feet together on a surface sloped at 18° and 26° in the frontal plane.

This weaker stability can be explained by biomechanical and neurophysiological factors. According to biomechanics, it is first necessary to consider the instability induced by the contralateral knee flexion, with articular freedom in rotations and varus-valgus movements given by knee flexion and not present during extension (Kapandji, 2011). In other words, stability may be compromised by an increased number of degrees of freedom. In addition, the lower limb flexion is only likely to compensate for the height difference between the two lateral sides of the board. It does not affect the slope itself, which gives the gravity force an additional tangential component that has to be counterbalanced by friction forces to prevent lateral sliding. It is also necessary to take into account the limitation of ankle free play when it is placed in a close-packed position in flexion (contralateral side) when the slope equals 15°. Indeed, goniometric data showed a flexion angle of about 18° in RI15 and LI15 while the maximum range of motion equals 11.6° to 14.7° (Soucie et al., 2011). Higher values than those reported in the literature for dorsiflexion may be explained by the experimental condition, which involved a closed-chain configuration in an upright, weight-bearing posture. In this condition, ankle flexion results from the action of the tibia, facilitated by body weight. In contrast, the open-chain configuration used in the literature, where the movement is performed by a therapist (Soucie et al., 2011), may produce less forceful motion.

In those conditions, the passive articular tension could significantly break the stabilizing ankle strategy (Horak and Macpherson, 1996). In addition, it has to be kept in mind that weight-bearing asymmetry, attested by Y0 variations in all lateral slope conditions, impairs body balance (Anker et al., 2008). As explained in a previous study (Atsawakaewmongkhon et al., 2024), the inclination of the support surface reduces the length of the base of support, which is equal to the foot length multiplied by the cosine of the slope angle (according to the trigonometric rule in a right-angled triangle). As a result, sloped surfaces are intrinsically less stable, with a reduced safety margin before reaching the limits of stability.

According to neurophysiology, the reorganization of posture, and especially of the lower limbs’ angular position, would require new programming of the postural muscles’ pattern of activity, as seen in the EMG recordings. In addition, the sensory inputs might also be more difficult to interpret with the changes in muscular length, especially the signals of Sol spindles that are stretched by ankle flexion. Previous studies have attested that the vibration of TA and Sol, which simulates the stretching of the muscle, results in postural reactions (Eklund, 1972). In the same way, the tangential component of gravity, which induces shear forces applied at the foot sole, might create a mismatch in the use of mechanoreceptors signals to assess the location of the CP at the foot surface. As a reminder, mechanoreceptors are widely distributed over the foot sole (Strzalkowski et al., 2018) and contribute to body balance under certain conditions (Meyer et al., 2004). According to the early work of Gurfinkel et al. (1995), maintaining body balance involves two complementary mechanisms: an operative system responsible for compensating deviations from a reference position, and a conservative system dedicated to setting the activity of anti-gravity postural muscles. Reorganizing lower-limb joint positions and muscle activity is likely to challenge the conservative system, whereas the difficulty of interpreting multisensory inputs on sloped surfaces may reduce the efficiency of the operative system. Consequently, both the ability to maintain the geometrical configuration of the new posture and the capacity to keep it balanced may be compromised.

The findings presented in the current study on the effects of support surfaces sloped in the frontal plane might have many implications in the field of ergonomics. First is the adaptation of lower limbs musculo-skeletal function which consists of a triple hip/knee/ankle flexion on the opposite side of the slope which requires a higher activity of rectus femoris and tibialis anterior. Second is an increase of postural sway associated with a CP shift toward the side of the slope, and then closer to the limit of stability. As a consequence standing on sloped surfaces in a professional context with extended work periods might increase the risk of musculo-skeletal disorders of the lower limbs and the risk of falls. This might be especially challenging for work at height, as in roofing activities.

While the experimental protocol used in this study enabled a detailed analysis of postural balance, complemented by goniometric and EMG assessments across varying support base slopes, several limitations should be acknowledged. First, the sample consisted exclusively of healthy young adults; therefore, age-related or pathological differences in balance control may not be represented. Second, the controlled laboratory conditions do not fully replicate real-world scenarios, where individuals may carry asymmetric loads (e.g., a bag or a tool), perform concurrent upper-limb tasks, or stand on surfaces inclined in oblique planes. Hence, the mechanisms underlying adaptation to sloped surfaces in the frontal plane during real-life activities remain to be fully understood.

## 5 Conclusion

The present study showed that support surfaces inclined in the frontal plane result in a CP shift toward the direction of the slope and in an increased postural sway, which both impair balance. The adaptation mechanisms, involving a triple flexion and a new muscular pattern of the contralateral lower limb, only partly compensate for the height difference between the two sides of the support surface. These findings could have useful applications in the field of ergonomics, particularly for fall prevention among workers in the construction industry.

## Data Availability

The original contributions presented in the study are included in the article/supplementary material, further inquiries can be directed to the corresponding author.
